# L-Type Ca^2+^ Channel Regulation by Calmodulin and CaBP1

**DOI:** 10.3390/biom11121811

**Published:** 2021-12-02

**Authors:** James B. Ames

**Affiliations:** Department of Chemistry, University of California, Davis, CA 95616, USA; jbames@ucdavis.edu; Tel.: +1-530-752-6358

**Keywords:** calmodulin, CaBP1, CaV1.2, CaV1.3, L-type Ca^2+^ channel, EF-hand, IQ-motif

## Abstract

L-type voltage-gated Ca^2+^ channels (CaV1.2 and CaV1.3, called CaV) interact with the Ca^2+^ sensor proteins, calmodulin (CaM) and Ca^2+^ binding Protein 1 (CaBP1), that oppositely control Ca^2+^-dependent channel activity. CaM and CaBP1 can each bind to the IQ-motif within the C-terminal cytosolic domain of CaV, which promotes increased channel open probability under basal conditions. At elevated cytosolic Ca^2+^ levels (caused by CaV channel opening), Ca^2+^-bound CaM binding to CaV is essential for promoting rapid Ca^2+^-dependent channel inactivation (CDI). By contrast, CaV binding to CaBP1 prevents CDI and promotes Ca^2+^-induced channel opening (called CDF). In this review, I provide an overview of the known structures of CaM and CaBP1 and their structural interactions with the IQ-motif to help understand how CaM promotes CDI, whereas CaBP1 prevents CDI and instead promotes CDF. Previous electrophysiology studies suggest that Ca^2+^-free forms of CaM and CaBP1 may pre-associate with CaV under basal conditions. However, previous Ca^2+^ binding data suggest that CaM and CaBP1 are both calculated to bind to Ca^2+^ with an apparent dissociation constant of ~100 nM when CaM or CaBP1 is bound to the IQ-motif. Since the neuronal basal cytosolic Ca^2+^ concentration is ~100 nM, nearly half of the neuronal CaV channels are suggested to be bound to Ca^2+^-bound forms of either CaM or CaBP1 under basal conditions. The pre-association of CaV with calcified forms of CaM or CaBP1 are predicted here to have functional implications. The Ca^2+^-bound form of CaBP1 is proposed to bind to CaV under basal conditions to block CaV binding to CaM, which could explain how CaBP1 might prevent CDI.

## 1. Introduction

### 1.1. Voltage-Gated L-Type Ca^2+^ Channel Structure and Function

Synaptic transmission and neuronal excitability are regulated by the L-type voltage-gated Ca^2+^ channels (CaV1.2 and CaV1.3, called CaV) expressed in the brain and heart [1,2,3,4]. CaVs display slow voltage-dependent gating characteristics (L-type) and are sensitive to a number of different dihydropyridine (DHP) antagonists and agonists [5]. Under resting basal conditions, intracellular Ca^2+^ concentration is kept low (100 nM) due to the powerful action of Ca^2+^ pumps and exchangers [1,6] and Ca^2+^ sequestration into stores [1,7]. The opening of CaV channels causes intracellular Ca^2+^ levels to increase into the micromolar range [8]. This Ca^2+^ influx triggers a wide range of Ca^2+^-dependent processes including gene transcription [9], neurotransmitter release [10], neurite outgrowth [11], and the activation of Ca^2+^-dependent enzymes [12]. Prolonged elevation of intracellular Ca^2+^ levels is cytotoxic [13], and CaV channels are negatively regulated by a process known as Ca^2+^-dependent inactivation (CDI) [14,15,16]. Dysregulation of CaVs are linked to various types of neurological disorders, including epilepsy, migraine, and chronic pain [17].

**Figure 1:**
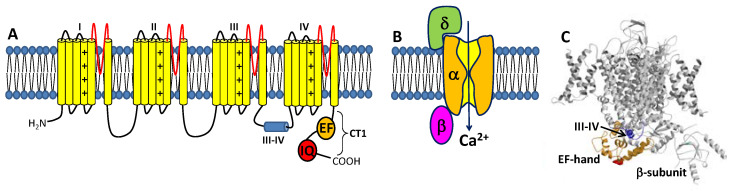
Structure of CaVs. (**A**) The α-subunit consists of 4 transmembrane domains (I-IV) that contain 6 helices (yellow) and pore loop (red). The III-IV linker is the inactivation gate. The cytosolic C-terminal domain (CT1) is comprised of an EF-hand domain (orange) and IQ-motif (red). (**B**) CaV is composed of pore-forming α-subunit attached to β- and δ-subunits. (**C**) Cryo-EM structure of CaV1.1 (PDB ID: 5GJW) showing the inactivation gate (III-IV linker in blue) connected to the EF-hand domain (orange). The IQ-motif is structurally disordered and missing in the cryo-EM structure of CaV1.1.

The CaVs are a heteromultimeric protein complex formed by a pore-forming α-subunit and regulatory β and δ subunits (Figure 1). The α-subunit contains four major transmembrane domains (Figure 1A), each with six membrane-spanning helices (termed S1–S6) and a positively charged S4 segment that controls voltage-dependent activation [18]. The transmembrane domains are connected by long cytoplasmic linkers (III-IV inactivation gate [19]), bracketed by cytoplasmic N-terminal and C-terminal domains [20]. The C-terminal domain (residues 1508-1665, called CT1) is important for Ca^2+^-dependent regulation of channel function and contains important sites (EF-hand and IQ motifs) for protein–protein interactions [21,22,23]. A three-dimensional structure of the skeletal muscle CaV (called CaV1.1) in the absence of CaM was solved by cryo-EM (Figure 1C) [24,25]. The CaV1.1 structure reveals long-range contacts between the inactivation gate (III-IV linker) and the channel EF-hand domain (orange in Figure 1C), which may undergo Ca^2+^-induced conformational changes during CDI (see Section 3 below).

**Figure 2:**
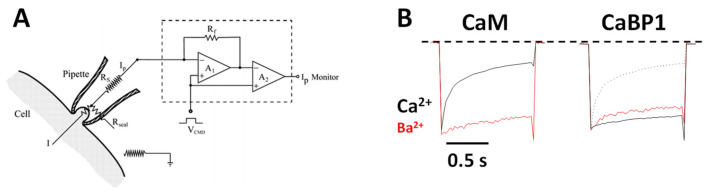
Ca^2+^-dependent Inactivation (CDI) of CaV. (**A**) Schematic representation of the electrophysiology experiment used to record CDI. (**B**) Normalized Ca^2+^ and Ba^2+^ currents evoked by 1 s pulse (−80 to +10 mV). Adapted from [2]. Fast decay of Ca^2+^ current due to CaM (black trace in left panel, CDI). The decay of the Ca^2+^ current is much slower in the presence of CaBP1 (black solid trace in the right panel, CDI abolished). Dotted line is the Ca^2+^ current in the absence of CaBP1, caused by endogenous CaM. Red traces are Ba^2+^ currents that lack fast inactivation because Ba^2+^ does not bind to CaM.

CaV channels inactivate rapidly by a process known as CDI (Figure 2) that depends critically on CaM [16,26] and CaBP1 [27,28]. Ca^2+^-free CaM is believed to be pre-associated with the CT1 domain such that the C-lobe of CaM interacts with the ‘‘IQ’’ domain and the N-lobe may interact with the EF-hand in order to increase the channel open probability under basal conditions [29,30,31]. Membrane depolarization causes CaV channel opening, which promotes a rise in intracellular Ca^2+^ that causes a conformational change in the CaV/CaM complex and gives rise to rapid channel inactivation called CDI [29,32,33,34]. CaBP1 competes with CaM for binding to CT1 [2,35], which prevents channel pre-association of CaM and abolishes CDI (Figure 2B).

### 1.2. CaM Is a Ca^2+^ Sensor for CaVs 

CaM is a 16.7 kDa Ca^2+^ sensor protein that belongs to the EF-hand superfamily [36]. CaM contains four EF-hand motifs (EF1, EF2, EF3, and EF4) that are grouped into two domains that are separately folded (EF1 and EF2 form the CaM N-lobe, while EF3 and EF4 form the CaM C-lobe) [37]. The CaM C-lobe and N-lobe each bind to Ca^2+^ with a dissociation constant of ~1 μM and 10 μM, respectively [38]. Thus, Ca^2+^ binding to CaM is an ordered process in which two Ca^2+^ bind to the C-lobe first before binding to the N-lobe. The Ca^2+^-bound form of CaM is known to bind to hundreds of different target proteins, including dozens of enzymes, receptors, ion channels, and other Ca^2+^ transporters [39]. The Ca^2+^-induced binding of CaM to its various target proteins usually serves to augment the biological activity of the target protein. 

**Figure 3:**
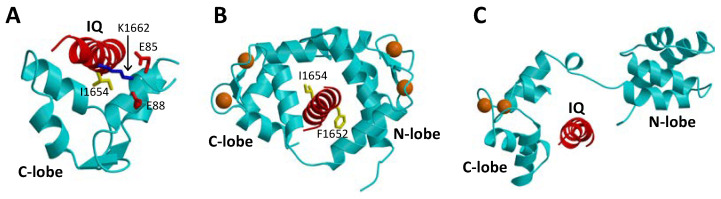
Atomic-level structures of CaM and CaBP1. (**A**) NMR structure of Ca^2+^-free CaM C-lobe (cyan) bound to the CaV1.2 IQ-motif in red (PDB ID: 6CTB) [41]. (**B**) Crystal structure of Ca^2+^-bound CaM (cyan) bound to the CaV1.2 IQ-motif in red (PDB ID: 2BE6) [44]. (**C**) Structural model of the crystal structure of CaBP1 (PDB ID: 3OX6) [45] bound to the CaV1.2 IQ-motif (red). Bound Ca^2+^ are indicated by orange spheres.

The binding of CaM to CaVs is critically important for promoting CDI [16,26]. In particular, CaM has been shown to bind to the IQ-motif (residues 1640–1665, highlighted red in Figure 1A) within the C-terminal cytosolic domain of CaVs [40], because deletion of the IQ-motif prevents CaV binding to CaM [26]. The NMR structure of Ca^2+^-free CaM (apoCaM) bound to the IQ-motif reveals that the IQ peptide forms an α-helix that interacts solely with the CaM C-lobe, while the IQ helix does not interact with the apoCaM N-lobe (Figure 3A). The most prominent intermolecular contacts involve IQ residues I1654 and K1662, and the mutations I1654E and K1662E each weaken apoCaM binding by nearly 10-fold [41]. The crystal structure of Ca^2+^-bound CaM bound to the IQ-motif reveals that both CaM lobes bind to opposite sides of the IQ helix (Figure 3B). The CaM C-lobe forms hydrophobic contacts with IQ residues I1654 and Q1655 that are essential for binding [42], hence the name IQ-motif. The CaM N-lobe forms hydrophobic contacts with aromatic IQ residues (Y1649 and F1652) that are essential for N-lobe binding. CaV mutations in the IQ-motif (I1654E and I1654M) that weaken CaM binding abolish CDI [43]. Much is known about how CaM interacts with the IQ-motif, but less is known about how the CaM-IQ interaction leads to channel inactivation. In this review, I present the possible molecular mechanisms of CDI to suggest how conformational changes in CaM and CaV might lead to CDI.

### 1.3. CaBP1 Promotes Activation of CaVs 

Neuronal Ca^2+^-binding proteins (CaBP1-5 [46]) represent a sub-branch of the CaM superfamily [39] that regulate various Ca^2+^ channel targets. Multiple splice-variants and isoforms of CaBPs are localized in different neuronal cell types [47,48,49] and perform specialized roles in signal transduction. CaBP1, also termed caldendrin [50], has been shown to modulate the Ca^2+^-sensitive activity of L-type channels [51], and the transient receptor potential channel, TRPC5 [52]. CaBP1 contains four EF-hands, similar in sequence to those found in CaM [39]. By analogy to CaM [37], the four EF-hands are grouped into two domains connected by a central linker that is four residues longer in CaBP1 than in CaM. In contrast to CaM, the first and second EF-hands of CaBP1 lack critical residues required for high affinity Ca^2+^ binding [46]. CaBP1 binds Ca^2+^ only at EF3 and EF4, whereas it binds Mg^2+^ at EF1 that may serve a functional role [53]. In addition to binding Ca^2+^, CaBP1 also binds tightly to the CaV IQ-motif [35]. A crystal structure is known for CaBP1 with Ca^2+^ bound to EF3 and EF4 (Figure 3C) [45]. A structural model of CaBP1 bound to the IQ-motif (Figure 3C) was generated here by homology modeling that was calculated based on the crystal structure of the CaM-IQ complex [44]. In this model, the Ca^2+^-bound CaBP1 C-lobe makes hydrophobic intermolecular contacts with IQ residues I1654 and Y1657, whereas the CaBP1 N-lobe does not make any intermolecular contacts. Future structural and mutagenesis studies of CaBP1 bound to the IQ-motif are needed to test the validity of the structural model in Figure 3C.

The binding of CaBP1 to CaV has been shown to increase the channel open probability and to abolish or prevent CDI. Unlike CaM, CaBP1 appears to cause CaV channel activation at high cytosolic Ca^2+^ levels, which gives rise to CaV channel CDF [45]. CaBP1 has been suggested to bind to multiple sites within CaV [54]; however, CaBP1 binding to the IQ-motif is believed to cause CDF [55]. The CaBP1 binding to the IQ-motif under basal conditions [35] may serve to block CaM binding to CaV, which may explain how CaBP1 prevents CDI. Schematic mechanisms are presented below to speculate how CaBP1 binding to CaV might activate channel open probability and prevent CDI.

## 2. CaV Channel Function Regulated by CaM and CaBP1

### 2.1. CaM Is Both an Accelerator and a Brake for CaV Channel Activity

**Figure 4:**
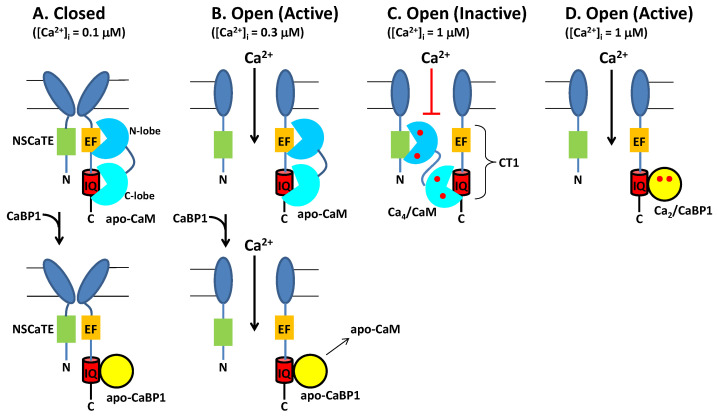
Conventional Model of CDI from CaV regulated by CaM and CaBP1. (**A**) Under resting conditions ([Ca^2+^]_i_ = 100 nM), CaV (dark blue) is in the closed channel state, which has been suggested to be pre-associated with Ca^2+^-free forms of CaM (cyan) or CaBP1 (yellow). (**B**) Membrane depolarization causes channel opening, which causes Ca^2+^ influx. Initially at low cytosolic Ca^2+^ levels (([Ca^2+^]_i_ < 300 nM), CaV is bound to Ca^2+^-free forms of CaM or CaBP1, which stabilize the active open state. (**C**) After sufficient Ca^2+^ influx, the cytosolic Ca^2+^ level increases to above 1 micromolar, which causes Ca^2+^ binding to CaM and the Ca^2+^-bound CaM promotes channel inactivation (CDI). Alternatively, CaV binding to CaBP1 (yellow) displaces CaM and prevents CDI (bottom panel). (**D**) The binding of Ca^2+^-bound CaBP1 to CaV promotes channel opening at elevated Ca^2+^ levels (called CDF). Bound Ca^2+^ are indicated by red circles.

Neuronal excitability is modulated in part by the Ca^2+^-dependent activity of CaV channels localized at the synaptic membrane. CaM binding to CaV serves to increase channel activity at low cytosolic Ca^2+^ levels under basal conditions ([Ca^2+^]_i_ = 100 nM). Conversely, CaM decreases CaV channel activity at higher cytosolic Ca^2+^ levels (([Ca^2+^]_i_ = 1.0 μM) caused by neuronal stimulation. Thus, CaM acts as both an accelerator and a brake to control CaV channel opening [16]. Ca^2+^ influx through CaV channels causes elevated intracellular Ca^2+^ levels that in turn promote a rapid negative feedback channel inactivation (called Ca^2+^-dependent inactivation or CDI [16]), mediated by CaM (Figure 4). Rapid CDI requires CaM to be pre-associated with CaV under basal conditions [29,33]. The channel has been suggested to be pre-associated with apoCaM under basal conditions (Figure 4A) [35], and apoCaM binding to CaV may increase Ca^2+^ currents (I_Ca_) and channel open probability (Po) [56], whereas I_Ca_ is dramatically decreased at elevated Ca^2+^ levels, because Ca^2+^-bound CaM inactivates the channel [15,16]. As a result, apoCaM binding to CaV in which the CaM C-lobe is bound to the IQ motif (red box in Figure 4) and CaM N-lobe is bound to the channel EF-hand (orange box in Figure 4) is believed to stabilize the channel in the open state at low Ca^2+^ levels under basal conditions (Figure 4B). At elevated Ca^2+^ levels (caused by neuronal stimulation), Ca^2+^-saturated CaM has been suggested to bind to the full-length CaV at two different sites: The N-lobe binds to the NSCaTE domain [15,57] and the CaM C-lobe binds to the IQ motif [44], which is hypothesized to stabilize the channel in the inactive state (Figure 4C). Atomic-level structures are known for Ca^2+^/CaM bound to IQ [44] and NSCaTE [57] domains. However, structures are not yet known for apoCaM and Ca^2+^/CaM each bound to the entire C-terminal cytosolic domain of CaV comprised of the channel EF-hand and IQ-motif (called CT1 domain, Figure 4C). Future studies are needed to elucidate the structural interaction of apoCaM and Ca^2+^/CaM each bound to the full-length channel to further test the model in Figure 4.

### 2.2. CaBP1 Binding to CaV Prevents CDI and Activates Channel Opening 

The upregulated expression of excess CaBP1 in particular neuronal cell types is known to abolish CDI of CaV [2,28,35,51] (Figure 4B, lower panel). The Ca^2+^-bound CaBP1 also increases CaV channel activity (Figure 4D) during Timothy Syndrome [58] and CaBP1 binding to CaVs could be targeted by therapeutics for the disease. CaBP1 was shown to compete with CaM for binding to the IQ-motif [35]. Thus, excess CaBP1 binds to the IQ motif and displaces apoCaM by mass action at low basal Ca^2+^ levels to prevent CaM-mediated CDI (Figure 4B, bottom panel). Previous studies have suggested that CaBP1 may bind to additional sites within CaV [54]. However, CaBP1 binding to the IQ-motif alone (as depicted in Figure 4) is believed to cause increased NPo under basal conditions and suppress CDI [28,55]. Future studies are needed to elucidate the atomic-level structural interactions between CaBP1 and CaV to further test the model in Figure 4.

## 3. Functional Role of Ca^2+^-Bound Forms of CaM and CaBP1 under Basal Conditions 

**Figure 5:**
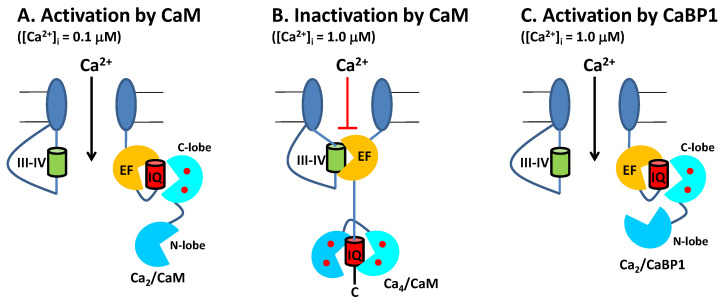
IQ-switch Model for CaV Regulation by Ca^2+^-bound forms of CaM and CaBP1. The channel EF-hand (orange) is proposed here to undergo a Ca^2+^-induced conformational change in which the channel EF-hand switches contact between the IQ-motif (red) and the III-IV linker (called IQ-switch). (**A**) Voltage-gated channel opening under resting conditions ([Ca^2+^]_i_ = 100 nM) generates CaV (dark blue) in the open channel state, which is pre-associated with CaM that contains a Ca^2+^ bound C-lobe (cyan) and Ca^2+^-free N-lobe (blue). In this activated open state of the channel, the IQ-motif is hypothesized here to be sandwiched between the channel EF-hand (orange notched circle) on one side and the Ca^2+^-bound CaM C-lobe on the other. (**B**) After sufficient Ca^2+^ influx, the cytosolic Ca^2+^ level increases to above 1 micromolar, which causes Ca^2+^ binding to both lobes of CaM that enables each CaM lobe to bind to opposite sides of the IQ helix (red) and promote channel inactivation (CDI). In the inactivated channel state, the channel EF-hand interacts with the III-IV linker (green helix called the inactivation gate) in place of the structurally related IQ-motif. A triple mutation in the channel EF-hand (T1591A/L1592L/F1593A) that disrupts interaction with the III-IV linker also abolishes CDI [43]. In essence, the channel EF-hand bound to the III-IV linker is proposed here to serve as a channel plug that blocks the channel entrance in the inactivated state (red bar in panel B). Channel opening is proposed to occur at low Ca^2+^ levels when the channel EF-hand engages the helical IQ-motif (stabilized by its binding to the CaM C-lobe), which disconnects the EF-hand from the III-IV linker to unblock the channel entrance (arrow in panel A). Therefore, Ca^2+^ binding to the N-lobe of CaM is hypothesized here to switch the channel EF-hand from interacting with the IQ-motif at low Ca^2+^ levels (active state in panel A) to interacting with the III-IV linker at high Ca^2+^ levels (inactive state in panel B) in order to promote CDI. (**C**) CaV binding to Ca^2+^-bound CaBP1 prevents CDI and promotes channel opening at elevated Ca^2+^ levels (called CDF). Ca^2+^-bound CaBP1 in panel C resembles CaM with two Ca^2+^ bound in panel A. Bound Ca^2+^ are indicated by red circles.

Previous Ca^2+^ binding studies reveal that Ca^2+^/CaM binds to the IQ-motif with a dissociation constant (K_d_ = 10^−12^ M [35,40]) that is a million times smaller than the K_d_ for apoCaM binding to the IQ [40]. The huge stabilization of Ca^2+^/CaM caused by IQ binding implies that CaM-IQ should bind to Ca^2+^ with much higher affinity than CaM alone. Indeed, on the basis of previous binding data [38,40], the apparent Ca^2+^ binding dissociation constant of the CaM C-lobe in the CaM-IQ complex (*K_D_^app^*) can be calculated to be ~100 nM ($K_{D}^{app}=\sqrt{\frac{1}{K_{1}K_{2}K_{3}}}$), where K_1_ = 10^12^ M^−2^ for the binding of two Ca^2+^ to the CaM C-lobe alone [38], K_2_ = 10^7^ M^−1^ for Ca^2+^/CaM C-lobe binding to the IQ-motif [40], and K_3_ = 10^−5^ M for the apoCaM C-lobe dissociation from the IQ peptide [40,41]. The predicted 100 nM binding of Ca^2+^ to the C-lobe of CaM-IQ implies that ~50% of the CaM-IQ complex should have Ca^2+^ bound to the CaM C-lobe under basal conditions ([Ca^2+^]_i_ = 100 nM, $K_{D}^{app}=100\;\mathrm{nM},$ and $Y=\frac{({\left[Ca^{2+}\right]}_{i}}{K_{D}^{app}+({\left[Ca^{2+}\right]}_{i}}=0.5$). By contrast, the CaM N-lobe in the CaM-IQ complex is estimated to bind to Ca^2+^ with an apparent dissociation constant ($K_{D}^{app}$) equal to ~1 μM ($K_{D}^{app}=\sqrt{\frac{1}{K_{1}K_{2}K_{3}}}$), where K_1_ = 10^10^ M^−2^ for the binding of two Ca^2+^ to the CaM N-lobe alone [38], K_2_ = 10^6^ M^−1^ for Ca^2+^/CaM N-lobe binding to the IQ [40], and K_3_ = 10^−4^ M for apoCaM N-lobe dissociation from the IQ [40]. This relatively low affinity Ca^2+^ binding to the CaM N-lobe predicts that the CaM N-lobe in the CaM-IQ complex should be devoid of Ca^2+^ under basal conditions. Therefore, I propose that a half saturated state of CaM with two Ca^2+^ bound to the C-lobe may exist under basal conditions ([Ca^2+^]_i_ = 100 nM) (see Figure 5A). This calcified CaM species would allow its Ca^2+^-bound C-lobe to be anchored tightly to CaV under basal conditions, which would enable tight CaM pre-association that is needed for rapid CDI. By contrast, the CaM N-lobe is predicted to be in the Ca^2+^-free state at basal Ca^2+^ levels that can switch to the Ca^2+^-bound state upon Ca^2+^ influx and serve as a Ca^2+^ sensor during CDI (Figure 5B). Future experiments are needed to measure the apparent Ca^2+^ binding affinity of CaM-IQ and CaM-CaV to experimentally verify whether the CaM C-lobe in these complexes can bind to Ca^2+^ with a $K_{D}^{app}$ near 100 nM as predicted above. Future studies are also needed to test whether Ca^2+^ binding to CaM is required to cause increased CaV channel open probability under basal conditions. In particular, the model in Figure 5 predicts that EF-hand mutations in CaM (that disable Ca^2+^ binding to the third and fourth EF-hands in the C-lobe) should prevent CaM pre-association, abolish CDI, and prevent the increased channel open probability observed under basal conditions [56]. Future electrophysiology and in vivo functional studies on CaV should be carried out in the presence of these CaM mutants to test the predictions in Figure 5.

A similar analysis performed here using the previous Ca^2+^ binding data for CaBP1 suggests that the binding of the IQ-motif to CaBP1 should allow the CaBP1-IQ complex to bind to Ca^2+^ in the nanomolar range, in contrast to the micromolar Ca^2+^ binding observed for CaBP1 alone [35,53]. The apparent Ca^2+^ binding dissociation constant of CaBP1 in the CaBP1-IQ complex (*K_D_^app^*) can be calculated to be ~100 nM ($K_{D}^{app}=\sqrt{\frac{1}{K_{1}K_{2}K_{3}}}$), where K_1_ = 10^12^ M^−2^ for the binding of two Ca^2+^ to CaBP1 alone [53], K_2_ = 10^9^ M^−1^ for Ca^2+^/CaBP1 binding to the IQ [35], and K_3_ = 10^−7^ M for apoCaBP1 dissociation from the IQ [35]. Future Ca^2+^ binding experiments are needed to verify whether the CaBP1-IQ complex (and CaBP1-CaV) can bind to Ca^2+^ in the nanomolar range as predicted here. The predicted 100 nM binding of Ca^2+^ to CaBP1-IQ implies that ~50% of the CaBP1-IQ complex should have two Ca^2+^ bound to CaBP1 under basal conditions ([Ca^2+^]_i_ = 100 nM, $K_{D}^{app}=100\;nM,\;$ and $Y=\frac{{\left[Ca^{2+}\right]}_{i}}{K_{D}^{app}+({\left[Ca^{2+}\right]}_{i}}=0.50$). Therefore, I propose that upregulated expression of CaBP1 in neurons will generate a Ca^2+^-bound form of CaBP1 that can exist at low Ca^2+^ levels under basal conditions as well as at high Ca^2+^ levels following Ca^2+^ influx (Figure 5C). Thus, Ca^2+^-bound CaBP1 is proposed here to constitutively activate CaV channels, which can both prevent CDI and promote CDF. Future experiments are needed to test whether Ca^2+^ binding to CaBP1 is required to cause increased CaV channel open probability. In particular, the model in Figure 5C predicts that EF-hand mutations in CaBP1 (that disable Ca^2+^ binding to the third and fourth EF-hands) should weaken CaBP1 binding to CaV, re-enable CDI, and prevent CDF. Future electrophysiology and in vivo functional studies on CaV should be carried out in the presence of these CaBP1 mutants to test the predictions in Figure 5C.

## 4. Concluding Remarks

CaV channels are oppositely regulated by CaM and CaBP1: CaM binding to CaV is essential for channel CDI, whereas CaBP1 binding prevents CDI and promotes CDF. A careful analysis of available Ca^2+^ binding data suggests that CaV binding to CaM (or CaBP1) causes a more than 10-fold increase in the apparent Ca^2+^ binding affinity of CaM (or CaBP1). Thus, a significant fraction of CaV channels are predicted to be bound to calcified forms of CaM (or CaBP1) under basal conditions ([Ca^2+^]_i_ = 100 nM vs *K_D_^app^* = 100 nM), which may have functional implications (Figure 5). Future studies on EF-hand mutants (that specifically abolish Ca^2+^ binding to either CaM or CaBP1) are needed to test whether Ca^2+^ binding to the C-lobe of CaM (or CaBP1) is essential for the increased CaV channel open probability caused by CaM (or CaBP1) under basal conditions.
